# Heavily Boron-Doped Silicon Layer for the Fabrication of Nanoscale Thermoelectric Devices

**DOI:** 10.3390/nano8020077

**Published:** 2018-01-30

**Authors:** Zhe Ma, Yang Liu, Lingxiao Deng, Mingliang Zhang, Shuyuan Zhang, Jing Ma, Peishuai Song, Qing Liu, An Ji, Fuhua Yang, Xiaodong Wang

**Affiliations:** 1 Engineering Research Center for Semiconductor Integrated Technology, Institute of Semiconductors, Chinese Academy of Sciences, Beijing 100083, China; mazhe@semi.ac.cn (Z.M.); 15680017025@163.com (Y.L.); denglx@semi.ac.cn (L.D.); zhangml@semi.ac.cn (M.Z.); shuy@semi.ac.cn (S.Z.); majing@semi.ac.cn (J.M.); pssong@semi.ac.cn (P.S.); liuqing@semi.ac.cn (Q.L.); jian@semi.ac.cn (A.J.); fhyang@semi.ac.cn (F.Y.); 2 College of Materials Science and Opto-Electronic Technology, University of Chinese Academy of Sciences, Beijing 101408, China; 3 School of Electronic, Electronical and Communication Engineering, University of Chinese Academy of Sciences, Beijing 101408, China; 4 State Key Laboratory for Superlattices and Microstructures, Institute of Semiconductors, Chinese Academy of Sciences, Beijing 100083, China; 5 School of microelectronics, University of Chinese Academy of Sciences, Beijing 101408, China

**Keywords:** heavily boron-doped silicon layer, boron etch-stop, thermoelectric, silicon nanowire, ZT, nanostructures

## Abstract

Heavily boron-doped silicon layers and boron etch-stop techniques have been widely used in the fabrication of microelectromechanical systems (MEMS). This paper provides an introduction to the fabrication process of nanoscale silicon thermoelectric devices. Low-dimensional structures such as silicon nanowire (SiNW) have been considered as a promising alternative for thermoelectric applications in order to achieve a higher thermoelectric figure of merit (ZT) than bulk silicon. Here, heavily boron-doped silicon layers and boron etch-stop processes for the fabrication of suspended SiNWs will be discussed in detail, including boron diffusion, electron beam lithography, inductively coupled plasma (ICP) etching and tetramethylammonium hydroxide (TMAH) etch-stop processes. A 7 μm long nanowire structure with a height of 280 nm and a width of 55 nm was achieved, indicating that the proposed technique is useful for nanoscale fabrication. Furthermore, a SiNW thermoelectric device has also been demonstrated, and its performance shows an obvious reduction in thermal conductivity.

## 1. Introduction

Structures at micro- and nanoscale introduce various new properties, which have become one of the most important research topics in recent decades. Actually, research into micro- and nanoscale structures has affected almost every field of our lives, including healthcare, biomedicine, information processing, etc. Thermoelectric materials can directly interconvert energies between heat and electricity based on the Seebeck and Peltier effects, which have no noise, no pollution, and extensive application prospects [1]. Compared with bulk silicon (Si) structures, one-dimensional Si nanowire (SiNW) possesses a higher Seebeck coefficient a lower thermal conductivity, and the thermoelectric figure of merit (ZT) value is several times higher [2,3,4]. Furthermore, Si is a potential thermoelectric material due to its low cost and the compatibility with complementary metal-oxide-semiconductor (CMOS) technology. It is of great significance to develop techniques with high reproducibility, high yield and excellent uniformity for fabricating a number of nanostructures with various shapes and dimensions.

For Si nanowires, there are many fabrication methods, and these can be divided into top-down and bottom-up approaches [5]. Bottom-up strategies refer to the process by which nanostructures are assembled by their atoms or molecules from a substrate. There are numerous routes for the bottom-up fabrication of semiconductor nanowires, such as the vapor-liquid-solid (VLS) method [6,7], solution-liquid-solid (SLS) method, and template-based synthesis [8,9,10]. Top-down fabrication strategies usually use etching to form nanostructures from a larger piece of material. Common methods in fabricating silicon nanowires include photolithography-related techniques and metal-assisted chemical etching processes [11,12]. For example, silicon nanowires can be mass produced with methods such as metal-assisted etching [13], the superlattice nanowire pattern transfer (SNAP) method [14] and the aqueous electroless etching (EE) method [15]. However, based on the premise of maintaining a high throughput, how to control the size remains a big challenge.

Recently, MEMS devices and processes have attracted increasing research interest, since they have provided us with integrated methods for silicon nanoscale structures fabrication. For instance, Toriyama et al. used electron beam lithography (EBL) to pattern silicon nanowires on separation-by-implantation-of-oxygen (SIMOX) wafer [16]. The silicon nanowire bridge (NWB) was released from the substrate by sacrificial layer etching. Koumela et al. fabricated silicon nanowire bridge with a diameter of 40 nm using a similar method [17]. Za’bah et al. reported a method for fabricating single crystal silicon nanowires with top-down optical lithography, together with simple oxidation and anisotropic etching with KOH [18]. However, all these approaches used silicon on insulator (SOI) wafer as the substrate, which is thirty times more expensive than a silicon substrate. The use of silicon substrate has also been considered by a number of authors. Tixier-Mita et al. obtained nanowires with a width of 100 nm by oxidation and tetramethylammonium hydroxide (TMAH) anisotropic etching of silicon [19]. Using oxide layer as a mask for KOH etching, Yang et al. achieved a 47 nm thick double-clamped beam on (111)-oriented silicon substrate [20]. However, both methods are complicated, and some specific silicon planes are required. 

Heavily boron-doped silicon layer and boron etch-stop techniques are usually used in the fabrication of MEMS devices [21,22,23]. However, in this paper, the authors introduce a method for fabricating nanoscale silicon structure based on these techniques. Boron etch-stop in TMAH solutions is utilized to release the silicon nanowire, which is easy to implement. A nanowire structure with a width of 55 nm is fabricated. Furthermore, metal electrodes were deposited by electron beam evaporation, and a thermoelectric device with a silicon nanowire of 150 nm in width is successfully finished. The device performances, including electrical conductivity, Seebeck coefficient and thermal conductivity, are also analyzed. 

## 2. Materials and Methods 

**Figure 1:**
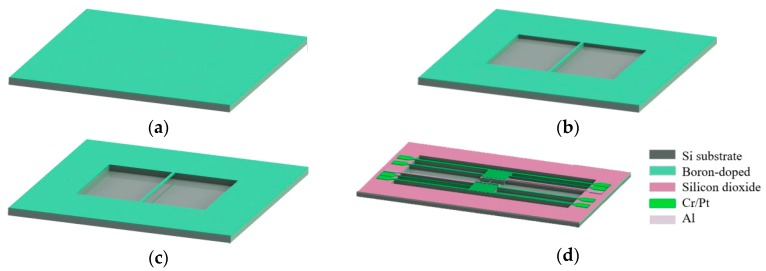
(**a**) Heavily boron-doped diffusion layer formed on the surface; (**b**) nanowire structure patterned by EBL and anisotropic ICP etching; (**c**) suspended Si nanowire released using TMAH solution; (**d**) schematic diagram of a thermoelectric structure using Si nanowire.

Figure 1a–c shows the schematic process flow of fabricating a Si nanowire structure using heavily boron-doped silicon layer, and Figure 1d shows the diagram of a thermoelectric structure using Si nanowire where the heating resistance and measuring electrodes are formed on both ends. In the experiment, 4-inch n-type, <100>-oriented, double-sided polished silicon wafers with a resistivity of 7–13 Ω·cm and thickness of 500 μm were used. Standard silicon cleaning steps were followed prior to the high-temperature diffusion process. To ensure a high figure of merit and the release of nanowire, silicon wafers were heavily doped by boron. The two-step boron diffusion was applied to obtain a heavily boron-doped layer. Pre-diffusion at 1050 °C lasting for two minutes with a solid-state B_2_O_3_ impurity source placed on both sides of the silicon wafer, and the drive-in diffusion under N_2_ at 1175 °C lasting for three minutes without an impurity source, were typically used (Figure 1a). During the pre-diffusion, solid-state B_2_O_3_ plates were placed on both sides of the silicon wafer, while the B_2_O_3_ plates were moved out for the drive-in diffusion. Several silicon wafers were placed on a quartz boat, with equal numbers of B_2_O_3_ plates laid in parallel in between. The distance between a wafer and a B_2_O_3_ plate was approximately 5 mm, in order to improve uniformity. The four-probe method was employed to measure the sheet resistance of the p-type-doped layer, which was 4.7 Ω/□, indicating that the surface impurity concentration was approximately 3.22 × 10^20^ cm^−3^, which sufficiently enabled self-etch-stop in TMAH solutions. Then, the silicon nanowire was defined with electron beam lithography (EBL) by a Raith150-Turnkey equipment (Dortmund, Germany) using electron beam (EB) resist ZEP 520 (Zeon Corporation, Tokyo, Japan). To obtain a steep side wall, inductively coupled plasma (ICP) etching by a Plasma-Therm VERSALINE DSE system (St. Petersburg, FL, USA) was employed, with an etching depth deeper than that of the heavily boron-doped layer (Figure 1b). The EB resist ZEP 520 was removed by acetone and then thoroughly rinsed in deionized water. Subsequently, wet etch was carried out with 5 wt % TMAH to remove the lightly doped silicon, which is beneath the heavily doped surface (Figure 1c). For the fabrication of the thermoelectric structure, a layer of silicon dioxide (SiO_2_) with thickness of 300 nm was grown by wet oxidation to isolate the substrate and metal electrodes. Photolithography was used to define the pattern of heating resistance and electrodes. Electron beam evaporation was used to deposit a stack of Cr (15 nm) and Pt (30 nm) on the surface, in which Cr acted as adhesion layer, and Pt acted as heating layer to generate a temperature gradient along the nanowire. Meanwhile, the Pt layer was also used as the temperature calibration resistance. Contact metallization and electrodes were then implemented with 500-nm-thick Al metal. A 2-μm-deep trench was etched to ensure electrical isolation of the nanowire from the substrate.

## 3. Results and Discussion

### 3.1. Diffusion Depth of Boron

In order to obtain a boron-doped layer with high quality, two-step boron diffusion was applied. A series of experiments was carried out at different diffusion temperatures and for different lengths of time. After optimizing the parameters, a homogenous and flat interface was obtained. Pre-diffusion and re-diffusion steps were conducted at constant temperature in a diffusion furnace, where N_2_ was constantly introduced to prevent it from contamination.

**Figure 2:**
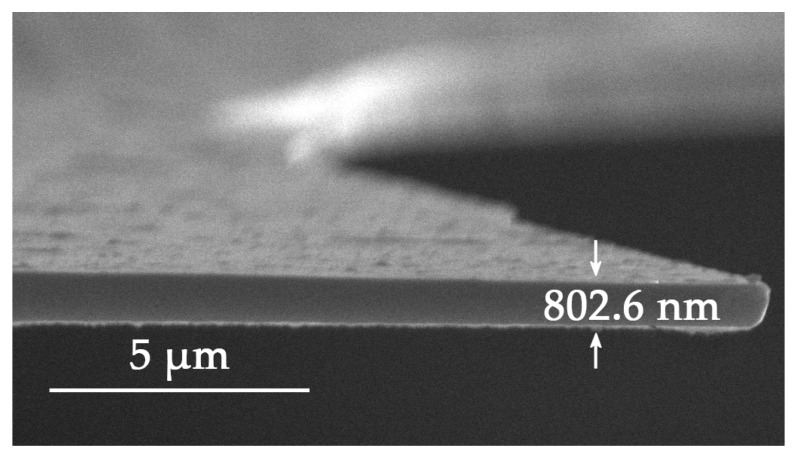
SEM image of the heavily boron-doped layer after TMAH etching, the thickness is about 800 nm.

To measure the thickness of the heavily boron-doped layer, the boron etch-stop technique in TMAH solutions was utilized, where the etch rate of heavily boron-doped silicon (more than 4 × 10^19^ cm^−3^) was much lower than that of the lightly doped layer. An etch rate of 0.01 μm/min was observed for a boron concentration of 2.5 × 10^20^ cm^−3^, while it was about 1 μm/min for a concentration lower than 1 × 10^19^ cm^–3^. The etching selectivity reached as high as 1:100 [23,24]. After being dipped in 5 wt % TMAH at 90 °C for 30 min, the heavily boron-doped layer was exposed, as shown in Figure 2. According to the scanning electron microscopy (SEM) image, for the sample with two minutes’ pre-diffusion at 1050 °C and three minutes’ drive-in diffusion at 1175 °C under N_2_ ambient, the thickness of the heavily boron-doped layer was approximately 800 nm. Figure 2 shows that the film surface was quite flat, and the thickness was quite even.

The average sheet resistance of the p-type-doped layer was 4.7 Ω/□, measured by the four-probe method. In addition, the corresponding impurity concentration was approximately 1 × 10^20^ cm^−3^, which satisfied the requirement for etch-stop selectivity of the boron-doped layer. Actually, in the subsequent procedures, it turned out that only a 20-nm-thick layer of heavily boron-doped silicon was etched by TMAH. 

### 3.2. Electron Beam Lithography and ICP

**Figure 3:**
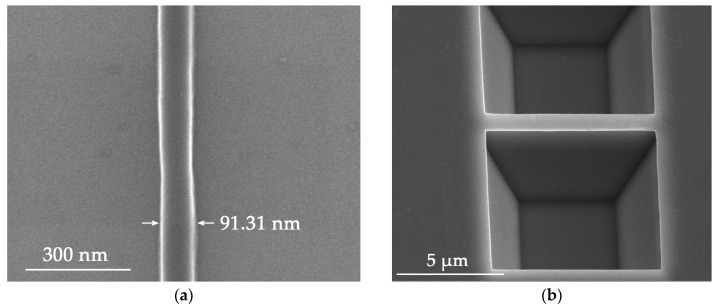
(**a**) Etch mask after the EBL; (**b**) nanowall after the ICP etching.

In order to obtain a feature size below 100 nm, EB resist ZEP 520 was employed as the etch mask for ICP. Electron beam lithography is suitable for nanoscale fabrication because of its excellent control precision. ZEP 520 is a common commercialized positive electron beam resist with the advantages of high resolution, vertical side wall and good etch resistance. In order to obtain nanowires with a good appearance, a group of experiments was carried out to optimize EBL parameters such as the resist thickness, the exposure dose and the line width. The resist was spun on the Si substrate with a thickness about 500 nm. The prebake was done under 180 °C for three minutes. Then, different wire widths were tried, as well as different doses of the electron beam. According to the design of the nanowire, the rectangular regions on both sides of the nanowire were exposed and developed. The nanowire was located in the middle and coated with resist. Figure 3a shows the details of the EBL patterned nanowire under high magnification, where the wire is about 90 nm in diameter, with a good line shape and smooth surface. These features indicate that the quality of the pattern is good enough for the following nanowire fabrication steps such as the ICP etching, where the resist will act as an etch mask.

Following the etch mask made by EBL, ICP etching was carried out in order to expose the substrate area under the boron diffused layer. ICP etching parameters with C_4_F_8_/SF_6_ of 40/30 sccm, and the Radio Frequency (RF) power of 800 W were optimized to obtain a smooth sidewall and small undercut. The final etch depth was about 1.6 μm, which exceeded the depth of heavily doped layer at 800 nm. However, during the experiments, the nanowall sometimes collapsed when the coated resist was 100 nm or less in width. One reason was that electron beam resist was overexposed to ensure a thorough exposure, so the resist was narrower than the layout geometry. The other came from the ICP process followed. The edge of the resist was etched first, resulting in narrower nanowires. Lateral undercut also reduced the width of nanowire. Therefore, these elements required an appropriate layout design from the beginning to avoid the width consumption when etching the nanowire. With a modified 900 nm layout geometry, a steep nanowall with a width of 670 nm was successfully fabricated, as shown in Figure 3b. It shows that the ICP process produced a very smooth surface of the sidewall.

**Figure 4:**
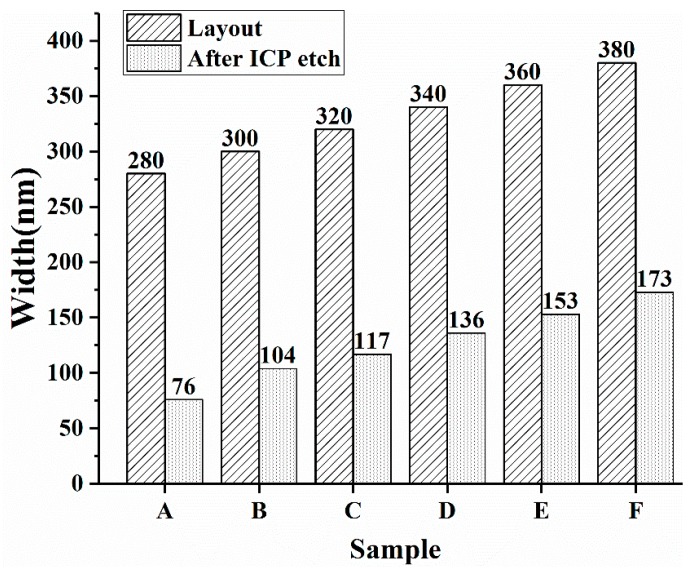
The comparison between the designed layout size and the nanowire width after the ICP etching.

Based on plenty of experiments, we the change of nanowire width can be observed in Figure 4. Data in samples A to F were obtained from our experiments, indicating that the ICP-etched nanowalls are approximately 200 nm narrower than the layout geometry, which provides a reference for manufacturing size-controllable nanowires.

### 3.3. Suspending of the Nanowire

**Figure 5:**
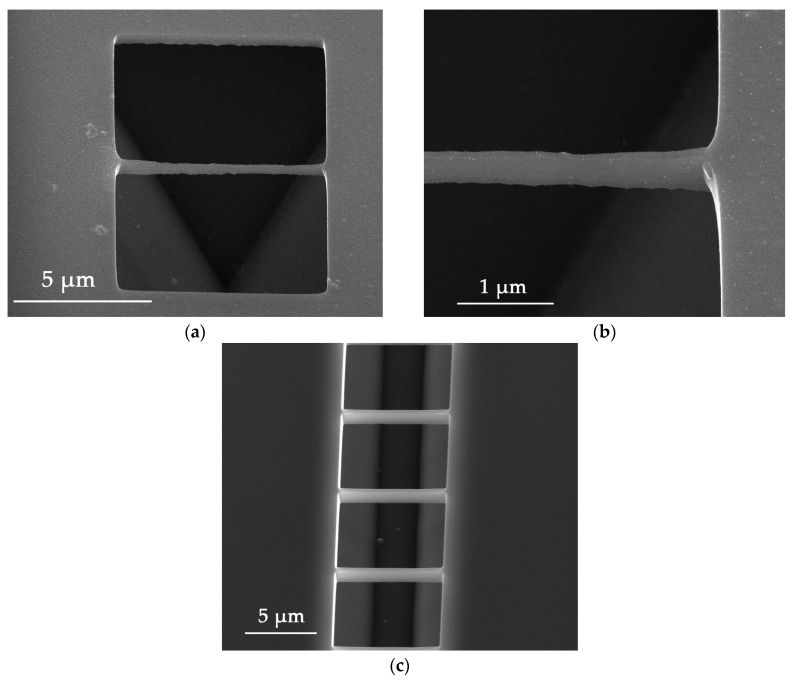
(**a**) Released nanowire structure with a length about 7 μm; (**b**) detail of the nanowire, the height is about 280 nm and width is about 55 nm; (**c**) simultaneously released nanowire array.

The bottom part of the nanowall has to be removed to obtain a suspended nanowire. After the ICP etching, the silicon nanowall structure was dipped into TMAH etching solution, which was able to etch the whole silicon wafer except the heavily boron-doped layer. This etch-stop technique definitely lowered the difficulty of making a suspended structure. According to Thong J.T.L. et al., the etch rate of a low-doped silicon wafer with (100)-orientation in 25 wt % TMAH reaches 1 μm/min at 80 °C, while it falls sharply for heavily doped silicon with a concentration greater than 4 × 10^19^ cm^−3^ [25]. When the boron-doping level reaches 1 × 10^20^ cm^−3^, the etching rate decreases to 10 nm/min for (100) facet, while the etch selectivity is up to 100 [23]. Hereby, for a 1020-nm-wide nanowall, it only takes about six seconds to etch through the bottom part. In fact, during our experiments, samples were etched in TMAH at 80 °C for three minutes to ensure the nanowire was able to be totally suspended. Figure 5a–c shows the released nanowall with different sizes. Figure 5a is a 7 μm long suspended structure, which shows a good uniformity, with 280 nm in height and 55 nm in width, as illustrated in Figure 5b. Experiments with 2- and 5-µm-long nanostructures were also successful, which indicates that nanowire structures with different lengths can be obtained by using the heavily boron-doped layer and its etch-stop character. For further demonstration, several parallel nanowire structures with the same length of 7 µm were released simultaneously. The result is shown in Figure 5c, where each nanowire shows almost the same etch effect such as the width and the height. Therefore, in the proposed method, nanostructures with regular shape and controllable size can be obtained. When this nanostructure is released in TMAH etch solution to obtain a suspended wire, the surface of the nanowire remains quite uniform. The height of the nanowire here is about 280 nm, which is a much larger value than the width, at 55 nm. However, it is reasonable to assume that a lower value could be obtained by shrinking the boron-doped layer thickness, which is our ongoing research work.

### 3.4. Fabrication of the Thermoelectric Device

After the nanowire structure was completed, bimetal filaments consisting of Cr/Pt were deposited on top of the sample as dual-purpose heaters/thermometers. Because platinum is sensitive to temperature change and exhibits a consistent temperature coefficient of resistance (TCR) within a certain temperature range, it was used as a heating and sensing filament. Due to the poor adhesion between Pt and silicon dioxide, a 15-nm-thick chromium layer was deposited firstly to enhance the adherence. The following procedure was depositing aluminum (Al) electrodes to make necessary electrical connections. Negative photoresist DNR-L300 is suitable for the metal lift-off process, because it can produce reversed trapezoid patterns, which is helpful for the lift-off of 500 nm Al. After the exposure and the development, a thin layer of photoresist probably still remained at the deposition area, which could cause the metal to peel off. To avoid this, samples were also dipped in 10% HCl for 10 s to remove the residual photoresist. Then the metal pads were thickened for wire bonding. Before electrical measurements were performed, 2-μm-deep trenches were etched to ensure electrical isolation of the nanowire from the substrate. 

**Figure 6:**
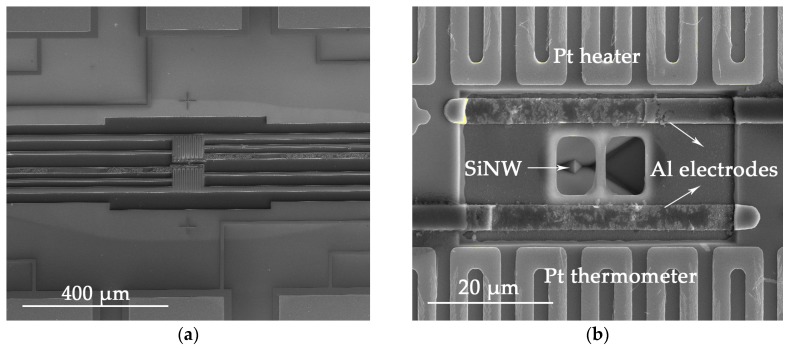
(**a**) SEM image of the device; (**b**) Nanowire suspended between two Pt serpentine resistances with Al metal electrodes.

Images of the device are shown in Figure 6. Figure 6a presents a SEM image of the finished device structure, which includes connecting wires and two bimetal filaments deposited at both ends of the nanowire. Figure 6b shows the nanowire suspended between Pt serpentine resistances, with the two ends connected via Al metal electrodes, prior to the isolation trench etching process. Al metal electrodes were used for *I*-*V* measurements. It can be seen that Pt filaments are smooth and uniform. The line width of Pt filament is about 3 µm, and the occupied area is about 70 × 90 µm^2^. The isolation trench is clearly shown in Figure 6a. 

### 3.5. Characterization of the Thermoelectric Device

In this work, a 7.66 µm long nanowire with the height of 685 nm and width of 408 nm was selected for the demo thermoelectric device structure. It has been known that ZT value is the most important parameter to indicate the efficiency of a thermoelectric device, with

(1) $$ZT=\frac{S^{2}\sigma }{k}T$$

where *S* is the Seebeck coefficient, *σ* is the electrical conductivity, *k* is the thermal conductivity and *T* is the temperature. To understand the thermoelectric performance of this device, the electrical conductivity, Seebeck coefficient and thermal conductivity have to be measured separately. In our work, all the measurements were carried out under vacuum conditions up to 10^−2^ Pa, and the environment temperature was kept at room temperature, 295.1 K.

The nanowire was connected to a Keithley 2450 source meter (Beaverton, OR, USA) to measure the *I*-*V* characteristics using the four-wire method, which eliminated the lead and the contact resistance [26]. The measured resistance by fitting the *I*-*V* curve of the sample was 357.14 Ω. Since the resistance is defined as

(2) $$R=\frac{L}{\sigma S_{a}}$$

Here, *L* represents the length of nanowire, σ the electrical conductivity and *S*_a_ the cross-sectional area of nanowire. By substituting the measured data to equation (2), the calculated electrical conductivity σ is 7.67 × 10^5^ S/m, which means the resistivity is 1.30 × 10^−3^ Ω·cm. So the corresponding doping concentration is 8.92 × 10^20^ cm^−3^. As we already know, the resistivity calculated from the sheet resistance 4.7 Ω/□ is about 3.22 × 10^−4^ Ω·cm. For the nanowire, the resistivity from the *I*-*V* curve is larger than that from the sheet resistance of the boron-doped layer. One possible reason is that the surface roughness of the nanowire exists although it is very small. The other reason comes from the oxidation of the nanowire surface during the process. Before the Pt serpentine resistances and Al metal electrodes were deposited, a 300-nm-thick silicon dioxide layer was grown on the sample surface by the wet oxidation process, which consumed 132 nm of boron-doped silicon. In addition, the surface of the silicon was etched by TMAH solutions. Using an etch rate of 0.01μm/min and an etching time of 10 min, about a 100-nm-thick area of silicon was removed.

The Seebeck effect causes a thermoelectric voltage by the temperature difference between the two ends of the material. The Seebeck coefficient *S* is defined as

(3) $$S=\frac{\Delta V}{\Delta T}$$

**Figure 7:**
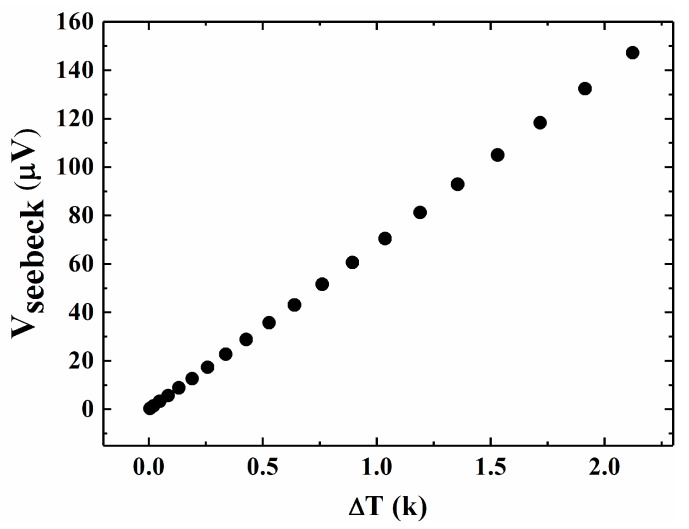
Seebeck voltage as a function of temperature difference.

In Equation (3), ΔT is the temperature difference and ΔV is the Seebeck voltage. *S* is in the unit of μV/K. It was measured as follows. There were two bimetal filaments deposited at both ends of the nanowire, one served as heating resistor and the other served as a thermometer [27]. Constant current was applied on the resistor to generate the heat. Part of the heat was transmitted to the other end of the nanowire, causing a temperature difference (ΔT), which was indicated by the change in resistance [28]. Meanwhile, a Seebeck voltage (ΔV) was induced [29,30], which was measured by a Keithley 2182A meter (Beaverton, OR, USA). The measurement was conducted at constant temperature to eliminate the influence of temperature variation of the atmosphere. Figure 7 shows the temperature dependence of the Seebeck voltage. By fitting the data, the Seebeck coefficient is 69.12 μV/K. As reported in Ref [14], for a Si nanowire with 50 nm in diameter and doping concentration of 1 × 10^20^ cm^−3^, the Seebeck coefficient reaches 100~120 μV/K, which is approximately two times larger. The main reason for this is probably that the diameter of the nanowire is not small enough to produce quantum confinement effects, which have an obvious influence on the Seebeck coefficient. Another reason is that the temperature difference between the two Pt filaments is larger than that of the nanowire, which results in smaller Seebeck coefficient.

Thermal conductivity was measured by the 3ω method, which is a typical approach for characterizing the thermal conductivity of one dimensional nanostructural materials [27,31]. Details of the measurement will be reported elsewhere. For the nanowire in this work, the calculated thermal conductivity is 43.37 Wm^−1^·K^−1^, which is obviously lower than bulk silicon (~150 Wm^−1^·K^−1^). As we already know, if the nanostructure has one or more dimensions smaller than the mean free path (MFP) of phonons but larger than that of electrons, the thermal conductivity would show a significant decline due to boundary scattering. The mean free paths of electrons and phonons at room temperature are 110 and ~300 nm, respectively. However, both the length and thickness of our specimen are larger than 300 nm, which might contribute little to the reduction of thermal conductivity. For doped nanowires, phonon-impurity scattering might also cause a reduction of thermal conductivity. When the doping concentration is one order of magnitude larger, only a small decrease of *κ* is observed [15]. If the doping level is 4 orders of magnitude larger, as in our case, a higher rate of phonon-impurity scattering may occur and result in a significant reduction in *κ*. In addition, for silicon nanowires fabricated by etching methods, the roughness at the nanowire surface is nonnegligible, since surface roughness would also introduce an extra form of scattering. This may also contribute to the reduction of the thermal conductivity.

Using Equation (1), the ZT of the demonstrated thermoelectric device is calculated to be about 2.49 × 10^−3^. This value is lower even than that of heavily boron-doped bulk silicon as reported, where the ZT value is about 0.01 [32]. By carefully analyzing our thermoelectric device structure and results, we found that the lower ZT value is mainly caused by the smaller Seebeck coefficient and larger thermal conductivity. Our measured Seebeck coefficient is about 3 times lower than other works [14]. As stated above, with reduced nanowire diameter and thickness, it is possible to produce quantum confinement effects [33], leading to a larger Seebeck coefficient. Meanwhile, multiple scattering mechanisms would lead to a smaller thermal conductivity for nanowires with smaller cross-sectional areas. The inaccuracy in temperature gradient measurement may also result in a lower ZT. The actual temperature gradient along the nanowire may be much smaller than the value measured by the Pt serpentine filaments, because the heat dissipates into the substrate when transmitting through the nanowire. Therefore, the ZT is likely to rise even higher by reducing the nanowire cross-sectional area and measuring the heat loss accurately.

## 4. Conclusions

In this paper, a top-down process using heavy boron-doping has been demonstrated for fabricate silicon nanowire and thermoelectric devices. The nanowire was defined by electron beam lithography and fabricated with ICP etching. TMAH etching was a critical step to suspending the nanowire from the substrate, and a 7-μm-long nanowire structure with a height of 280 nm and a width of 55 nm was obtained. Multiple measurements were carried out to characterize the electrical conductivity σ, the Seebeck coefficient S, and the thermal conductivity κ of the device. The ZT was calculated to be 2.49 × 10^−3^, resulting from a small S and large κ. By reducing the nanowire cross-sectional area, the thermal conductivity is likely to reduce significantly; thus, a larger ZT could be expected.
